# Non-noble, efficient catalyst of unsupported α-Cr_2_O_3_ nanoparticles for low temperature CO Oxidation

**DOI:** 10.1038/s41598-017-14779-x

**Published:** 2017-11-01

**Authors:** Ali Bumajdad, Shaimaa Al-Ghareeb, Metwally Madkour, Fakhreia Al Sagheer

**Affiliations:** Chemistry Department, Faculty of Science, Kuwait University, P.O. Box: 5969, Safat, 13060 Kuwait

## Abstract

Herein, we report the synthesis of chromium oxide nanoparticles, α -Cr_2_O_3_ NPs, followed by full characterization via XRD, SEM, XPS, and N_2_ sorptiometry. The synthesized nanoparticles were tested as catalysts toward the oxidation of CO. The impact of calcination temperature on the catalytic activity was also investigated. CO conversion (%), light-off temperature, T_50_, data were determined. The results revealed that chromia obtained at low calcination temperature (400 °C) is more active than those obtained at high calcination temperatures (600° or 800 °C) and this is ascribed to the smaller particle size and higher surface area of this sample. The results revealed a superior catalytic activity of Cr_2_O_3_ NPs at lower temperature as we reached a complete conversion at 200 °C which is high value in the forefront of the published results of other non-noble catalysts. The high activity of Cr_2_O_3_ nanoparticles (T_50_ as low as 98 °C) where found to be dependent on a careful selection of the calcination temperature. These results may provide effective and economic solutions to overcome one of the major environmental threats.

## Introduction

The primary pollutants from vehicles comprised of carbon monoxide (CO), hydrocarbons (HCs) and nitrogen oxides (NOx)^1^. These three harmful pollutants are major source of air pollution and it affects humans, vegetation, and atmosphere in number of ways. Among all types of exhaust gases carbon monoxide is most harmful^2^. Carbon monoxide is an odourless, colourless and toxic gas. It is also called the silent killer and its known to contributes indirectly to global warming and ozone depletion^3^. Thus, CO levels in the ambient air play a role in determining the air quality of a region. Noble metals are known for their high oxidation power and terms as paramount in automobile industry since the seventeenth century. So far, most of effective catalysts for this system have been reported to use the supported noble metals^4–9^. These catalysts exhibited high activities for CO oxidation; however, they have some disadvantages with a high cost, a limited availability and low selectivity at high temperatures. Although not as efficient as noble catalysts, some non-noble metal oxides (e.g. CeO_2_-based catalyst) show high activity for CO oxidation, and hence, still advantageous due to their lower cost. For example, the light-off temperature, T_50_, for a CoOx/CeO_2_ catalyst was reported to be 135 °C (i.e. 50% of CO converted to CO_2_ at that temperature)^10^. Reported also CuO-CeO_x_ hybrid ceria catalyst for CO oxidation and showed T_50_ around 94 °C^11^. Also, Fe-Cu/CeO_2_ composite catalysts were tested for CO oxidation and showed T_50_ around 158 °C^12^. Also, Co_3_O_4_@CeO_2_ core shell cubes with optimized CeO_2_ shell thickness exhibited 100% conversion of CO at 190 °C in CO oxidation^13^.

Recently, CO oxidation at low temperature with nonprecious metal based catalysts was an important research goal^14–18^. With a special focus to Cr_2_O_3_, very rare reports disclosed its usage for CO oxidation. Ghandhi *et al*.^19^ reported the CO oxidation using Cr_2_O_3_ and they obtained T_50_ values of 265 °C. Ren *et al*.^20^ reported the T_50_ values of 200 °C-pretreated mesoporous Cr_2_O_3_ at 151 °C and they found it to be higher than those of the corresponding 400 °C-pretreated Cr_2_O_3_ which was 147 °C.

The present work aimed to produce a non-noble metal catalyst (other than the extensively studied cerium oxide catalyst) with a high activity for the CO catalytic oxidation at lower temperature. Based on a literature survey, it is the first time to utilize nano chromia as a catalyst, without doping it with other metal oxide or using a support, for CO oxidation with such low-temperature efficiency. In this context, we prepared chromia nanoparticles via simple method at different calcination temperatures. Full characterization to the synthesized nanoparticles was investigated to stand on the most promising characteristics leading to efficient catalytic activity.

## Experimental part

### Preparation of chromia nanoparticles

25% aqueous ammonia solution was added dropwise to a continuously stirred 0.1 M aqueous solution of the nitrate salt of Cr. The resulting mixture was stirred for another hour, then was left overnight. The precipitate thus obtained was filtrated through a Wattman filter paper (No. 42), washed with double distilled water, and dried overnight at 100 °C. The dried precipitate was ground, sealed in vials, and stored over silica gel in a desiccator till further use. The dried precipitates were treated thermally by calcination on heating in a still atmosphere of air at various temperatures (400, 600, or 800 °C) for 2 h. The calcination products thus obtained are indicated below by corresponding oxide formula and an added Arabic numeral to symbolize the temperature applied.

### Characterization of Nanoparticles

The X-ray diffraction (XRD) measurements were conducted by using a Bruker AXS D8 Advance X-Ray Powder Diffractometer with a copper target and a nickel filter with Cu Kα radiation (λ = 0.154 nm). Measurements were performed in the range 20–80° (2θ). The morphology of the particles, as well as electron diffraction patterns were obtained by scanning electron microscopy (SEM) using a JEOL JSM-7001F operating at 120 kV.

X-ray photoelectron spectroscopy (XPS) surface elemental analysis was conducted using a model Thermo ESCA Lab 250xi equipped with Mg Kα radiation (1253 eV) and operated at 23 kV and 13 mA. The binding energy was referenced to the C 1s line at 284.76 eV for calibration. N_2_ adsorption-desorption isotherms were measured on test samples at liquid nitrogen temperature (−195 °C) using a model ASAP 2010 automatic Micromeritics sorptiometer (USA) equipped with a degassing platform.

### Catalytic activity measurements

A weighed portion (250–300 mg) of the test catalyst, was placed at ambient temperature on a G1-porous quartz disc mounted in the middle of a tubular reactor (i.d. = 2 cm; length = 15 cm) equipped with a tubular, sealed sheath for a Pt/Rh thermocouple compatible with a Type-J Cole-Parmer (USA) digital thermometer for reaction temperature reading accurate to within ±2 °C. The catalyst was pre-activated by *in situ* heating (at 20 °C min^−1^) in a 200 Torr portion of O_2_ gas at 200 °C for 15 min. The heating was enabled by a temperature-controlled OMEGA (USA) tubular furnace mounted around the reactor. Subsequently, the gas was pumped off to 10^−2^ Torr, and a fresh 200 Torr portion of O_2_ was admitted into the reactor and maintained for 15 min, prior to further outgassing. This process of gassing and outgassing of O_2_ at 200 °C was repeated for two more times, before a final outgassing at 200 °C and cooling to room temperature (RT). A 300 Torr portion of the reactants mixture (CO + O_2_ with a 1:3 mass ratio) was expanded into the reactor at RT, then temperature was increased at 20 °C min^−1^ to certain higher temperatures in the range 50–400 °C keeping an almost constant interval of ca. 50 °C. Each temperature was maintained for a 15-min period through which the gas samples were withdrawn from the reaction mixture (reactants plus products). It is worth noting, that the reactor hot zone was counter-parted by a cold zone (a refrigerated water jacket) maintained at 10 °C, adopting a design similar to that described previously by Schwab *et al*.^21^.

50-μl gas samples were withdrawn from the reaction atmosphere of catalytic CO oxidation by means of a Hamilton gas syringe at different reaction temperatures (three samples at each temperature) and analyzed by a model CP-9001 Chrompack gas chromatograph (The Netherlands). The GC was equipped with a TCD detector (maintained at 120 °C) and a packed column of PORAPACK Q (maintained at 110 °C). A 99.99% pure He (KOAC) was employed as the carrier gas (20 ml min^−1^), and an installed MOSAIC software facilitated a computer processing of the experimental results versus pre-constructed calibration curves. For accurate quantitative results, calibration procedures are required. Calibration involves the correlation between a known concentration of a component and the resultant detector signal generated when that component is detected. Electronic integrators are used to convert the detector signal to either peak area or height. Calibration algorithms are incorporated into the integrator or computer software used for data acquisition and analysis. Thus, one creates calibration tables in the data system through the use of prompted dialogs or menu items. The simplest procedure involves creating a single-level calibration and is achieved by the following process. First one optimizes the chromatographic parameters for the desired separation and identification of components (such as choice of column, detector, injection process, and oven temperature).

## Results and Discussions

### Characterization of chromia nanoparticles

**Figure 1:**
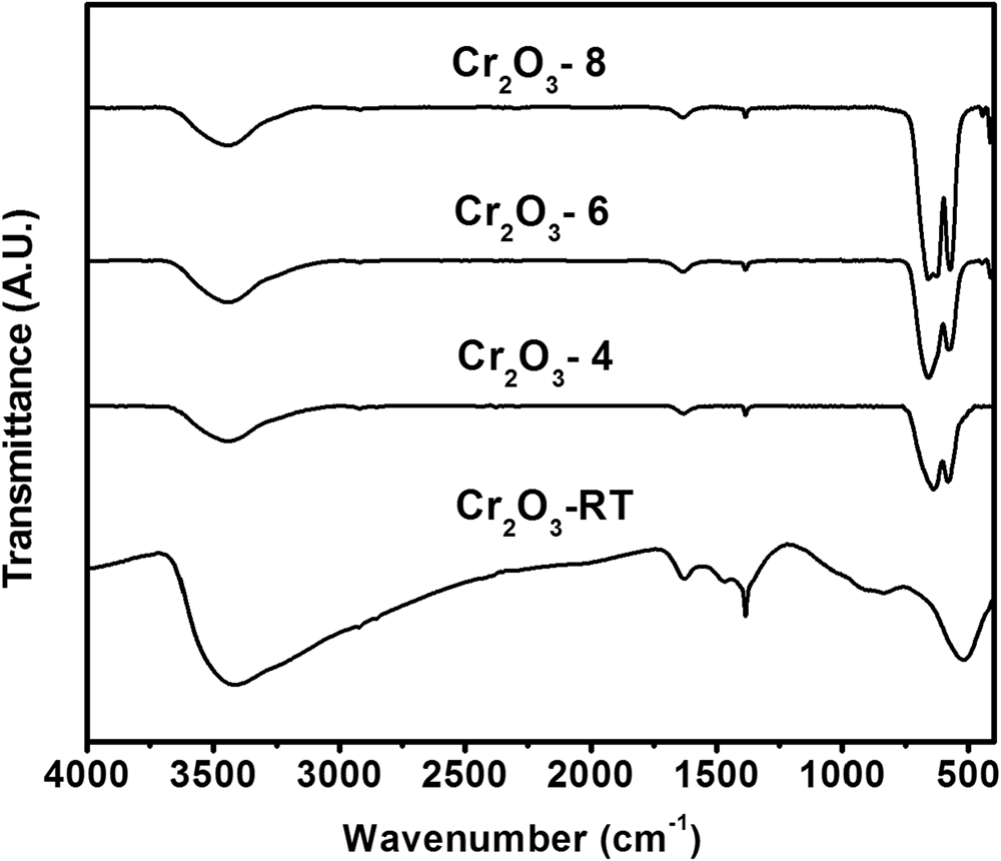
FT-IR spectra of the as-obtained Cr_2_O_3_ and its calcined products at 400°, 600° and 800 °C.

IR spectra obtained for the as-obtained chromia, Cr_2_O_3_-RT, and its calcination products at 400–800 °C are shown in Fig. 1. IR spectrum of the Cr_2_O_3_ sample displays a strong absorption at 523 cm^−1^, a broad shoulder at 839 cm^−1^, a sharp band at 1384 cm^−1^, a weak shoulder at 1468 cm^−1^ and a weak band 1628 cm^−1^. The spectrum also exhibits a strong and broad band centered around 3416 cm^−1^. The broad band shown at 3416 cm^−1^ is due to υO-H of H-bonded hydroxyl groups. The peak at 1628 cm^−1^ can be assigned to δH-O-H bending vibration of physically adsorbed water molecule. The band at 1467 cm^−1^ is attributed to υCOO of carbonate impurities species. The absorption at 1384 cm^−1^ is due to υNO_3_^−^ vibrations, which originate from the metal precursor. The bands below 1000 cm^−1^ is related to the υCr-O bands. IR spectra of the calcination products (at 400–800 °C), are significantly different from that of the as-prepared Cr_2_O_3_. The IR spectrum of Cr_2_O_3_-4 only exhibits two sharp, strong bands at 640 and 581 cm^−1^, in addition to a much weaker absorption at 3453 cm^−1^. Upon further increase of the calcinations temperature up to 800 °C two additional low-frequency bands emerge at 445 and 414 cm^−1^. The four absorption at 661, 574, 445 and 414 cm^−1^ are assignable to υCr−O lattice vibration of α-Cr_2_O_3_ crystallites^22^.

**Figure 2:**
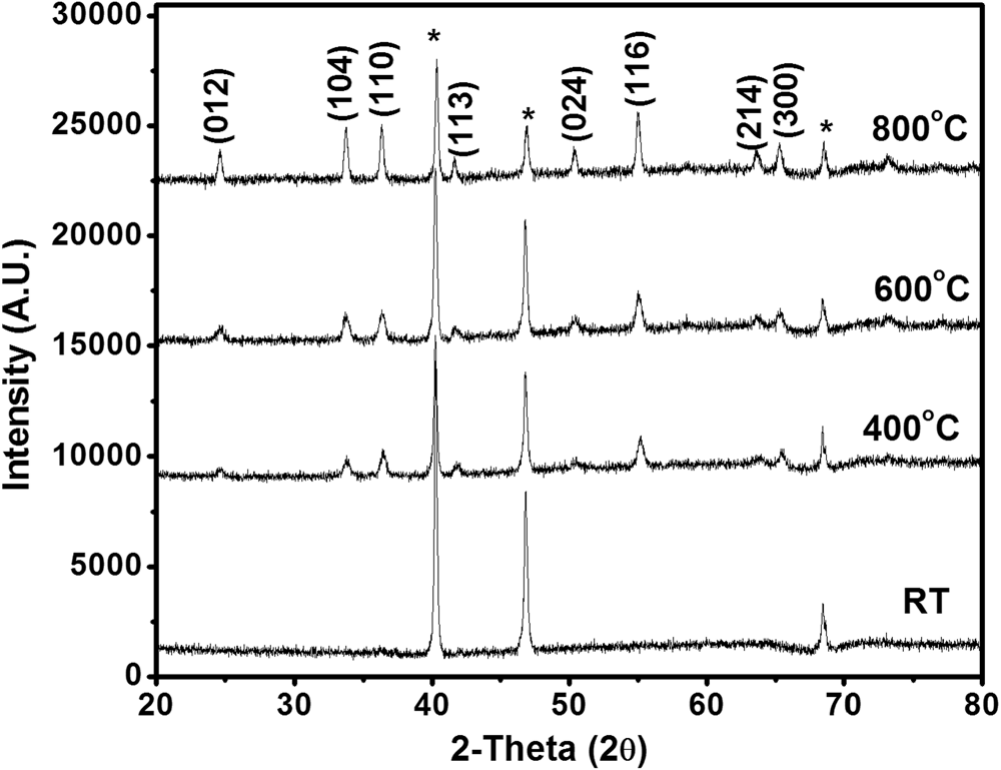
X-ray powder diffractograms for the as-obtained Cr_2_O_3_ and its calcination products (400°–800 °C). *-labelled peaks are due to the Pt/Rh sample holder.

XRD diffractogram recorded for the as-obtained Cr_2_O_3_ and its calcination products (400–800 °C) are exhibited in Fig. 2. It is obvious from the results that the as-obtained Cr_2_O_3_ is largely non-crystallite, which is reflected in its gelatinous nature. The calcination products are shown to be crystalline. The crystallinity is shown to increase with calcination temperature. In spite of that, the calcination products are shown to exhibit the same diffraction pattern, which is similar to that filed in JCPDS card no. 01-1294^23^ for α-Cr_2_O_3_. The crystal size can be calculated according to Debye-Scherrer formula:

1 $${\rm{D}}=k{\rm{\lambda }}/{\rm{\beta }}\,cos{\rm{\theta }}$$

where K, a shape factor, k = 0.89, λ is the wavelength of the Cu-Kα radiations, ß is the full width at half maximum and θ is the angle obtained from 2θ values corresponding to maximum intensity peak in XRD pattern. The mean crystallite size values were found to be an increase function of temperature and equal 36 nm, 40 nm and 56 nm for Cr_2_O_3_-4, Cr_2_O_3_-6 and Cr_2_O_3_-8 respectively.

**Figure 3:**
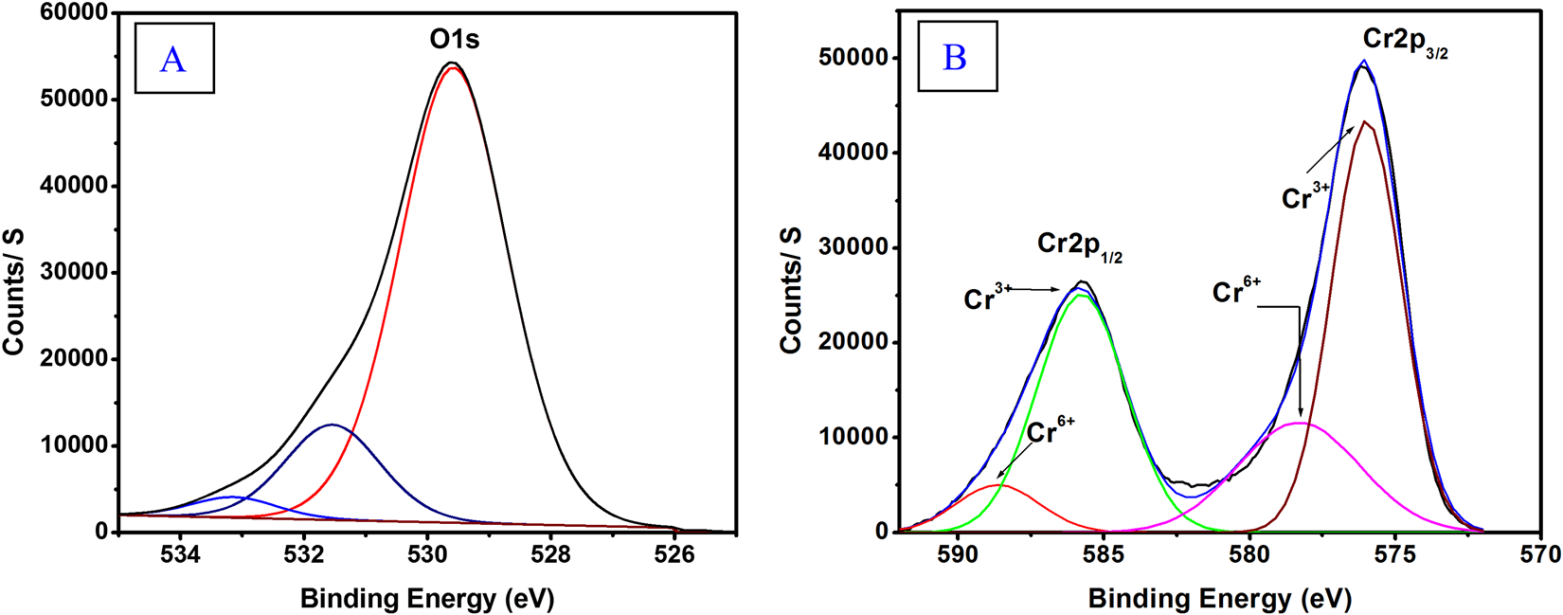
Deconvoluted XPS peaks of (**A**) O (1s) and (**B**) Cr (2p) and for Cr_2_O_3_-4.

XPS results obtained for a chromia, Cr_2_O_3_-4, are shown in Fig. 3. The binding energy of all the deconvoluted peaks of the studied samples are tabulated in Table 1. Cr(2p) electron binding energy values were found to be located at 586 and 587ev for Cr 2p_1/2_, and 576 and 579 eV for Cr 2p_3/2_ respectively. The lower binding energy peak located at 576 eV is assigned to the Cr^3+^ state^24^. While the peak located at 579 eV is attributed to higher oxidation state Cr^6+^ ^24^. The results indicate that despite the fact that the calcination products are essentially α-Cr_2_O_3_, the surface is partially oxidized to expose Cr^≥3+^ ions. Similar results have been reported by Fahim, *et al*.^25^. The Cr^≥3+^/Cr^3+^ surface atomic ratios, as were calculated from 2p XPS signal of Cr, are 0.18, 0.32, 0.42, 0.31 for Cr_2_O_3_-RT, Cr_2_O_3_-4, Cr_2_O_3_-6, and Cr_2_O_3_-8, respectively. The slight amount of surface OH^-1^ is due to the unavoidable physical water adsorption at the surface^26^. The O1s XPS spectrum showed three components with O1s binding energies at 529.7, and 531.1 eV, assigned to lattice oxygen and the peak at 533.2 eV is assigned to surface defects and/or adsorbed surface hydroxyls^27^. The O/Cr surface atomic ratio were found to be 4.6, 2.8, 2.1, and 2.1 for Cr_2_O_3_-RT, Cr_2_O_3_-4, Cr_2_O_3_-6, and Cr_2_O_3_-8, respectively. The higher ratio at low calcination temperature is expected and it is due to the formation of Cr_2_O_3_.nH_2_O structure (oxide-hydroxide structure). This is also evident from the presence of two type of O^2−^ signals shows in Table 1, the lower binding energy one’s is believed to be for surface lattice oxygen from the nonhydrated Cr_2_O_3_ formula while the higher bending energy is from water associated with the chromia formula (i.e. hydrated Cr_2_O_3_). The third peak at around 533 eV are corresponding to the OH^**−**^/H_2_O species physisorbed at the surface. In other words, beside the XPS signal of the lattice oxygen, there is two other signals resulted from two type of water interacting with the catalyst surfaces. One resulted from chemisorbed water encouraged by the presence of lattice defects/vacancies and the other is due to physisorbed water molecules experiencing dipole-dipole interactions with the lattice oxygen.

**Table 1:** XPS data for the as-obtained Cr_2_O_3_ and its calcination products (400°–800 °C).

Test catalyst	Surface composition		
	Emission	Species^#^	BE(eV)
Cr_2_O_3_-RT	O(1s)	O^2−^	529.6
	O(1s)	O^2−^	531.2
	O(1s)	OH/H_2_O	533.2
	Cr(2p3/2)	Cr^3+^	576.8
	Cr(2p3/2)	Cr^6+^	578.9
	Cr(2p1/2)	Cr^3+^	586.5
	Cr(2p1/2)	Cr^6+^	588.3
Cr_2_O_3_-4	O(1s)	O^2−^	529.9
	O(1s)	O^2−^	531.6
	O(1s)	OH/H_2_O	533.5
	Cr(2p3/2)	Cr^3+^	576.0
	Cr(2p3/2)	Cr^6+^	577.9
	Cr(2p1/2)	Cr^3+^	585.7
	Cr(2p1/2)	Cr^6+^	587.9
Cr_2_O_3_-6	O(1s)	O^2−^	529.8
	O(1s)	O^2−^	531.4
	O(1s)	OH^−^	532.9
	Cr(2p3/2)	Cr^3+^	576.2
	Cr(2p3/2)	Cr^6+^	578.3
	Cr(2p1/2)	Cr^3+^	585.9
	Cr(2p1/2)	Cr^6+^	588.1
Cr_2_O_3_-8	O(1s)	O^2−^	529.6
	O(1s)	O^2−^	531.5
	O(1s)	OH^−^	533.1
	Cr(2p3/2)	Cr^3+^	576.0
	Cr(2p3/2)	Cr^6+^	578.2
	Cr(2p1/2)	Cr^3+^	585.7
	Cr(2p1/2)	Cr^6+^	587.9

^#^The O^2−^ species are: for Cr_2_O_3_ (lower binding energy) and Cr_2_O_3_.nH_2_O (higher biding energy).

We believe that, the chemical composition of Cr_2_O_3_-4 sample play a major role in this, lowest reported, light-off temperature (98 °C) for undoped and unsupported chromia (i.e. the occurrence of the metal oxide surfaces of sample Cr_2_O_3_-4 at a transition intermediate between Cr_2_O_3_.nH_2_O ⇔ Cr_2_O_3_ play a major role in such low light-off value).

**Figure 4:**
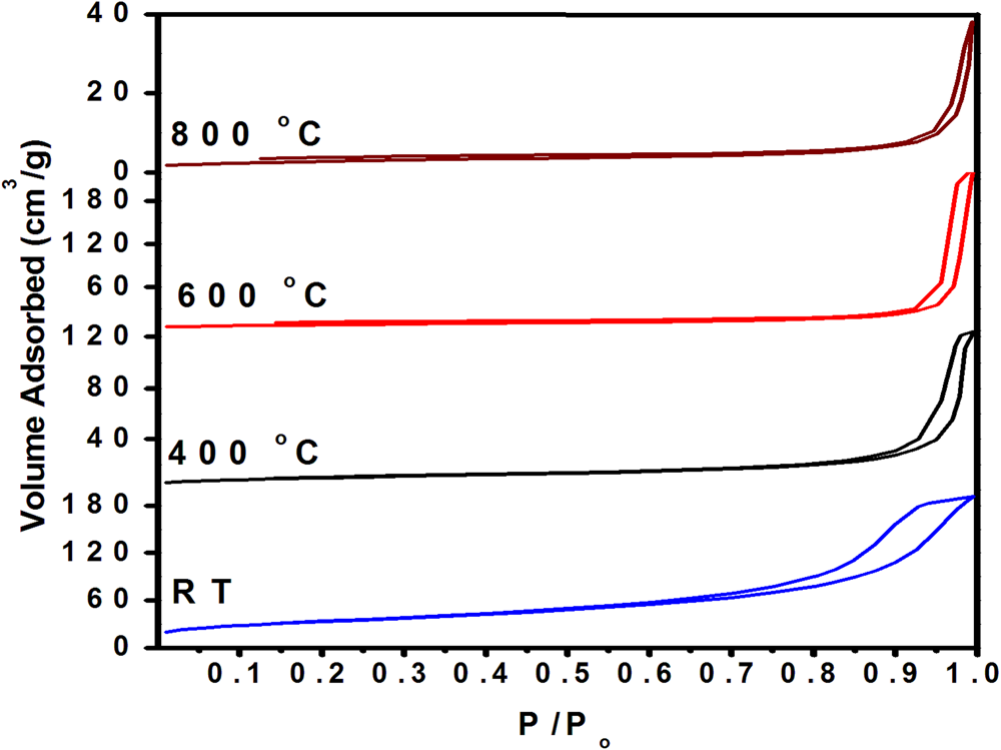
N_2_ adsorption-desorption isotherms for the as-obtained Cr_2_O_3_ and its calcination products.

N_2_ adsorption-desorption isotherms determined at −195 °C on the as-obtained Cr_2_O_3_ and its calcination products (400°−800 °C) are shown in Fig. 4. It is obvious from the classification reviewed elsewhere^28^, the isotherms obtained are of type-IV. Type-IV isotherms imply largely mesoporous surfaces. Except for the as-obtained α-Cr_2_O_3_, i.e., non-crystalline chromia, the hysteresis loop displayed has closure points at p/p_◦_ < 0.45 which may account for narrow mesopores. Hysteresis loops exhibited by as-obtained α-Cr_2_O_3_ and its calcination products (400°–600 °C) are of type-H2. Whereas α-Cr_2_O_3_-8 displays type-H3 loop. Type-H2 loop is believed to be associated with ink-bottle-like pores of varying radius, often generated by agglomerates or compacts of spheroidal particles of non-uniform size and arrangement. A type-H3 loop is attributed to adsorbate condensation in capillary spaces between parallel plates or open slit-shaped capillaries.

The S_B_ values observed for the as-obtained Cr_2_O_3_ is 127 m^2^/g. Upon calcination at 400°−800 °C, the S_B_ value decreased to 29 and further to 17 m^2^/g (see Table 2). A S_B_ ≈ S_C_ indicates presence of cylindrical mesopores while the result (S_C_ > S_B_) exhibited by Cr_2_O_3_-8 implies the presence of non-cylinderical mesopores. The Cr_2_O_3_ showed lowest pore volume value (0.0385 cm^3^/g) at 800 °C (Table 2), which is a direct result of the high temperature calcination and consequent sintering of particles. The as-obtained Cr_2_O_3_ itself shows the highest pore volume (0.235 cm^3^/g). Upon calcination (400°–800 °C) α-Cr_2_O_3_ particles are produced, which exhibit a higher *V* ^*c*^_*p*_ compared to *V*_*p*_ thus implying the presence of non-cylindrical mesopores (see Table 1). The as-obtained α-Cr_2_O_3_ shows narrower pore size than the calcined ones. As the calcination temperature increases from 400° up to 800 °C, a pore widening is observed (*r*_*p*_ **/**nm = 11 → 23 nm). Concomitantly, a drop in pore volume (0.0937 → 0.0385 cm^3^/g) is observed. This supports a pore widening mechanism (Table 2).

**Table 2:** BET surface area (S_B_/m^2^/g), -specific surface area (S_C_/m^2^/g), pore volume (*V*_*p*_(cm^3^/g) and radius (r_p_ (nm) and crystallite size (*L*/ ± 1 nm), for the as-obtained Cr_2_O_3_ and its calcination products at 400°–800 °C.

Material	*V*_*P*_/(cm^3^/g)	*r*_*p*_/nm	S_B_/(m^2^/g)	S_C_/(m^2^/g)	*L*/nm
Cr_2_O_3_-RT	0.235	4	127	130	0
Cr_2_O_3_-4	0.094	11	29	28	36
Cr_2_O_3_-6	0.075	14	25	23	36
Cr_2_O_3_-8	0.039	23	17	133	52

**Figure 5:**
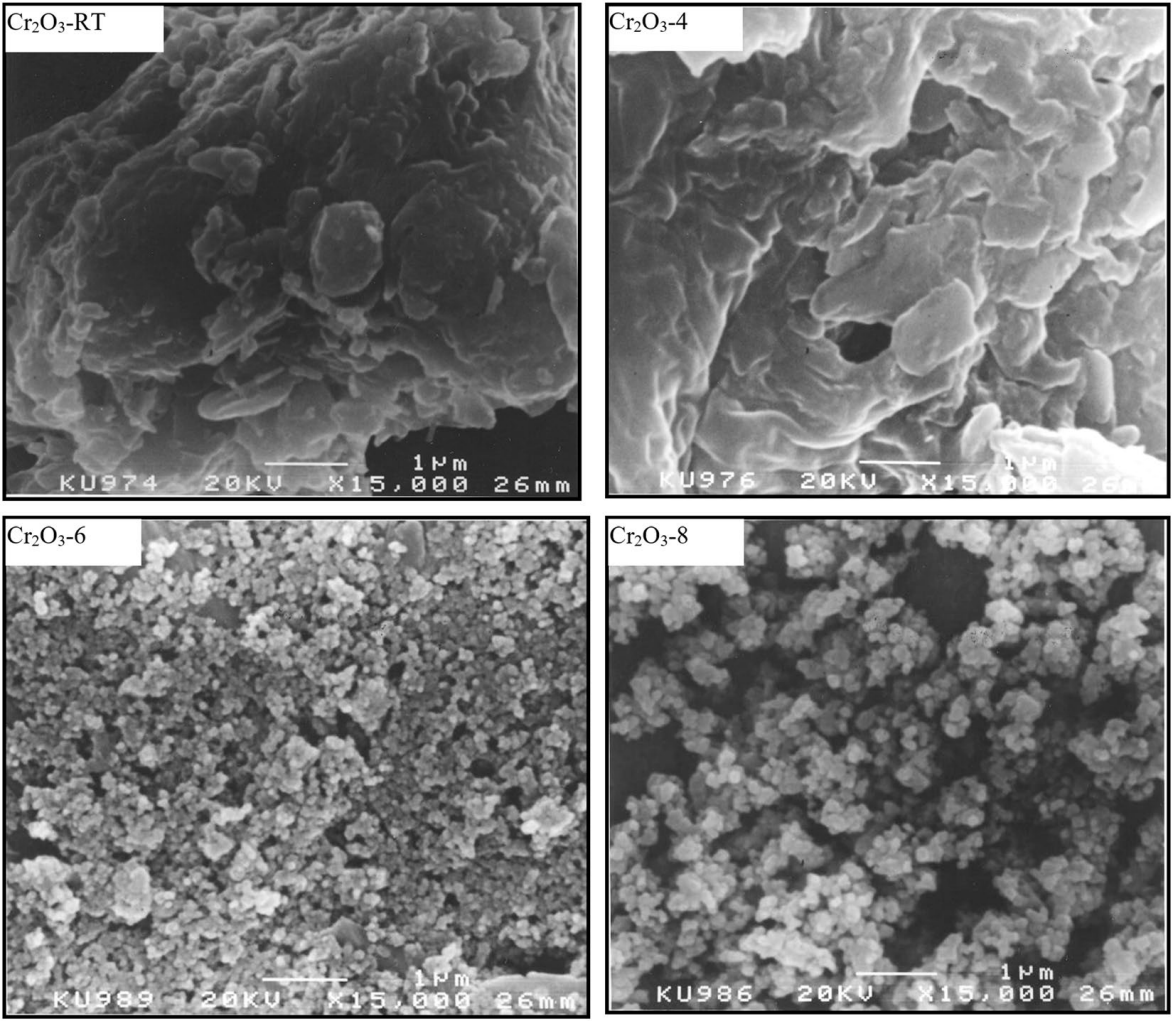
SEM micrographs for the as-obtained Cr_2_O_3_ and its calcined products at 400°, 600° and 800 °C.

SEM images obtained for as-obtained Cr_2_O_3_ and its calcination products at 400°−800 °C are shown in Fig. 5. SEM indicates that the as-obtained Cr_2_O_3_ and Cr_2_O_3_-4 products exhibit irregular clumps of particles of ill-defined contours, whereas the calcined samples at ≥600°C produce a different morphology characterized by uniform particle size showing quazi-spherical particles. It is also seen that the size of these particles increases with calcination emperature from 600° to 800 °C.

### CO oxidation activity

**Figure 6:**
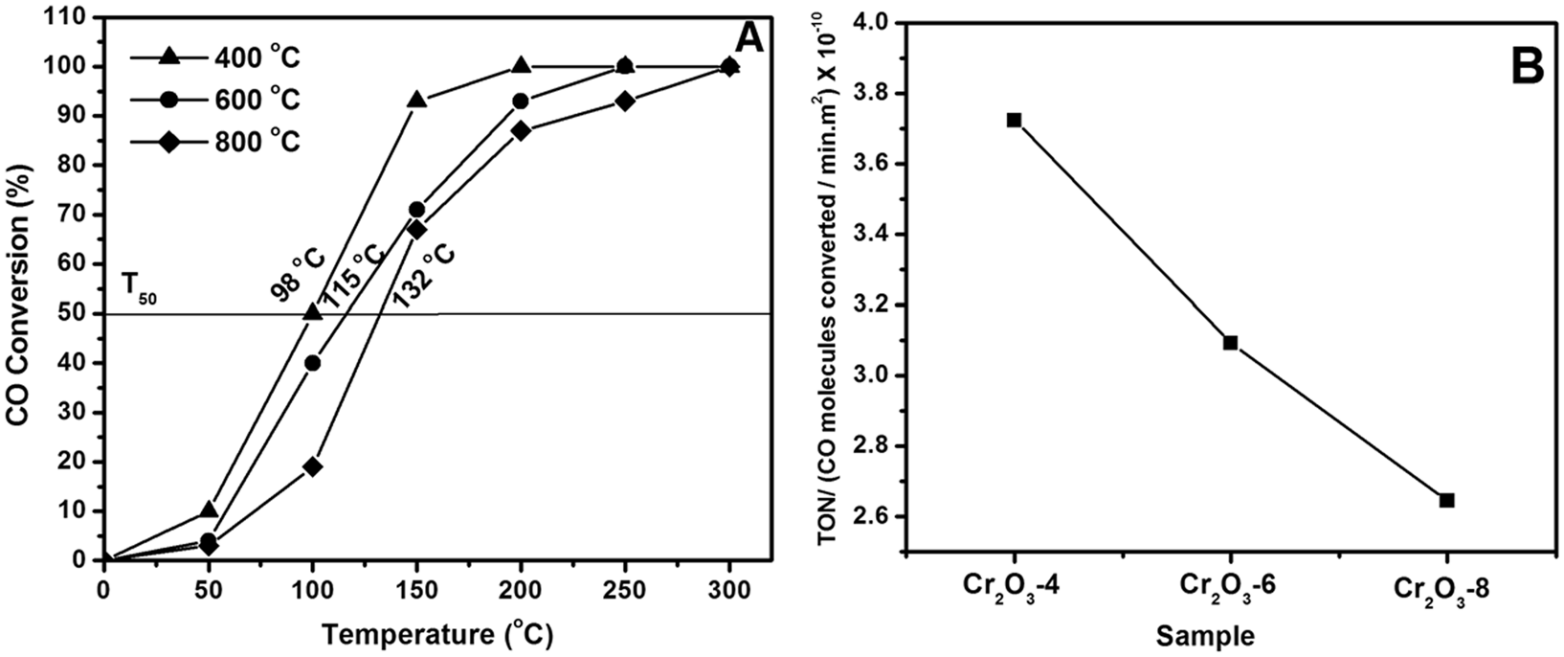
(**A**) Plots of CO conversion vs. reaction temperature for Cr_2_O_3_ nanoparticles at different calcination temperatures. (**B**) The Turn Over Number of the three-studied catalysts at 100 °C.

On chromia surfaces, redox couples of Cr^3+^/Cr^>+3^are important catalytic oxidation sites^29^. Figure 6A compares plots of CO conversion versus reaction temperature for chromia catalysts derived from Cr_2_O_3_ at various calcination temperatures.

The results brought about reveal that CO oxidation on the chromia catalysts occurs actively at low temperatures as shown in Fig. 6A. T_100_ is the temperature at which 100% of CO was oxidized and T_50_ is the light-off temperature and their corresponding values are represented in Table 3 in which the present results were compared with their corresponding non-noble catalysts reported in the literature. Our results also reveal that chromia obtained at low calcination temperature (400°C) is more active than those obtained at high calcination temperatures (600° or 800 °C). From the XPS data, we found that, the higher the calcination temperature, the lower the surface content of Cr^>+3^ sites. Since it is known that on chromia surfaces intimately coupled Cr^3+^-Cr^>+3^ sites facilitate the electron mobility required for CO oxidation, and, thus, considered the active sites^29^. Hence, the drop in activity upon increasing calcination temperature may be linked to the concomitant loss or excess changes on chromia surfaces. It is worth noting, that the three test samples of chromia are of comparable surface area, thus the surface area may not be a key parameter in shaping up the CO oxidation activity on the present test samples. Moreover, chromia is an acidic oxide, and, hence, surface basic sites are also of less importance to the surface catalytic activity in the present case.

**Table 3:** T_100_ temperature and T_50_ (the light-off temperature) for Cr_2_O_3_ at different calcination temperatures (400°–800 °C) and example of previously reported data on different simple, and multi-component oxides. References are indicated for more information about the catalysts composition, surface properties, crystallite phases and experimental/operational conditions.

Material	Experimental Conditions	*T*_*100*_, °C	T50, °C	[Ref.]
Cr_2_O_3_-4	Catalyst dose: 250–300 mg	200	98	Present
Cr_2_O_3_-6	CO + O_2_ with a 1:3 mass ratio	248	115	Present
Cr_2_O_3_-8		304	132	Present
Cr_2_O_3_	45–46 g (80 cm^3^) of catalyst, gas composition, CO~l.2%, O_2_~1.2′ %, balance He; gas rate-1400 cm^3^/min.	340	265	19
CuO/Cr_2_O_3_ on silica	The feed composition was 2% CO, 2% O_2_ in helium. 0.1 g of catalyst at a flow rate of 100 ml min^−1^	≈500	213	32
CuO/Cr_2_O_3_ on Alumina		≈320	233	32
Co_3_O_4_ hexagonal plates	50 mg of catalyst. The feed gas (1.6% CO, 21.0% O_2_, and balanced N_2_) at a total flow rate of 25 ml min^−1^	≈117	95	33
Co_3_O_4_ cubes		≈130	112	33
Co_3_O_4_ tetrakaidecahedrons		≈120	110	33
CuxCeO_2_-X	0.10 g of the catalyst. The reaction gas containing CO (2400ppm) and O_2_ (15 vol%) and balance Ar was fed through the catalyst bed at a rate of 100 mL/min.	253	186	34
CoxCeO_2_-X		300	216	34
CuMnOx	200 mg of catalyst (40–60 mesh). The standard composition of the feed gas was 1% CO, 20% O_2_, and 79% N_2_ with a space velocity (SV) of 20000 mL/(h·g_cat_).	140	35	35
CuO–CeO_2_	50 mg of catalyst. The reaction mixture consisted of 1 vol.% CO, 1.25 vol.% O_2_ and 50 vol.% H_2_ in He	200	94	36
Co_0.9_Fe_2.1_O_4_	20 mg catalyst were used. The reaction gas mixture consisted of 1% CO and 10% O_2_ in argon with a total flow rate of 15 mL/min	235	205	37
α-Fe_2_O_3_	20 mg of the catalyst. The total flow rate was 15 ml min^−1^ with 1% of CO and 10% O_2_	385	325	38
Co/CeO_2_	Catalyst dose: 250–300 mg CO + O_2_ with a 1:3 mass ratio	200	150	39

The results presented in Fig. 6B, are for the intrinsic (i.e. exclude the specific surface area from consideration) activity of the calcined catalyst. In other words, the graphs show the turn over number, TON, per CO molecules per min per m^2^. Accordingly, TON-based ranking of the test oxides shows that at 100 °C, the Cr_2_O_3_-4 samples exhibit a better intrinsic activity than Cr_2_O_3_-6 and Cr_2_O_3_-8. This means that, among the studied catalysts, Cr_2_O_3_-4 exhibits the best activity and 100 °C intrinsic activity. It is worth mentioning that, from the XPS results, the Cr^≥3+^/Cr^3+^ surface atomic ratios of Cr_2_O_3_-4 is 0.32 which is equal to that found for Cr_2_O_3_-8 (0.31) but less than that of Cr_2_O_3_-6 (0.42). This means that, for the studied samples, the Cr^≥3+^/Cr^3+^ surface ratio is not the only factor that control the intrinsic activity and other factors are playing roles. This also support our conclusion that the occurrence of Cr_2_O_3_-4 at the transition intermediate between Cr_2_O_3_.nH_2_O ⇔ Cr_2_O_3_ is the determining factor here. The different morphological structure of the Cr_2_O_3_-4 catalyst (see Fig. 5) may also play role in such superiority^30^.

Kinetic catalysis studies have shown the pathway adopted by CO oxidation on metal oxides to be dependent on the reaction temperature regime applied^31^: (i) at ≤150 °C, Eley-Rideal mechanism (CO_(g)_ + O_2_(ads) or O_2(g)_ + CO(ads), ∆E ≤ 10 kCal/mol; (ii) at 150–250 °C, Langmuir-Hinshelwood mechanism (CO(ads) + O_2_(ads)), ∆E ca. 10–20 kCal/mol; and (iii) at ≥250 °C, Mars-van-Krevelen mechanism (CO_(g)_ + O(lattice)), ∆E ≥ 20 kCal/mol. Based on the studied temperature, the Langmuir–Hinshelwood mechanism is expected (both the reactants (CO and O_2_) are adsorbed on the catalyst surface and O_2_ get activeated and reacted with the CO. The involvement of lattice oxygen is rolled out since 100% CO conversion is reach at relatively low temperature (200–300 °C). The four elementary steps of the Langmuir-Hinshelwood mechanism of CO oxidation. with the following elementary reaction Eqs (2–5):

2 $${1/{\rm{2O}}}_{{\rm{2}}}+{\rm{X}}\leftrightarrow {\rm{O}}\cdot {\rm{X}}$$

3 $${\rm{CO}}+{\rm{X}}\leftrightarrow {\rm{CO}}\cdot {\rm{X}}$$

4 $${\rm{O}}\cdot {\rm{X}}+{\rm{CO}}\cdot {\rm{X}}\to {{\rm{CO}}}_{{\rm{2}}}\cdot {\rm{X}}+{\rm{X}}$$

5 $${{\rm{CO}}}_{{\rm{2}}}\cdot {\rm{X}}\leftrightarrow {{\rm{CO}}}_{{\rm{2}}}+{\rm{X}}$$

where “X” denotes the active site.

## Conclusion

In this study, we reported a new CO oxidation catalyst of Cr_2_O_3_ nanoparticles prepared via hydrothermal technique. The effect of calcination temperature on the crystal structure and composition was investigated via XRD and XPS measurements. The catalytic activities of Cr_2_O_3_ calcined at different temperature were determined and revealed superior activity (T_50_ as low as 98 °C) was achieved for Cr_2_O_3_-4 samples that occur at the intermediate composition transition between Cr_2_O_3_.nH_2_O ⇔ Cr_2_O_3_ (i.e. hydrated to nonhydrated chromite transition). We attributed this superior activity to this chemical composition nature in addition to its other surface characteristics. The results of this study will make its applicability to be economically possible in automotive exhausts and factory chimneys.
